# Evaluation of the cobas MTB and MTB-RIF/INH assay in clinical samples for the detection of *Mycobacterium tuberculosis* in respiratory specimens

**DOI:** 10.1128/jcm.01959-24

**Published:** 2025-03-25

**Authors:** Ulrich Eigner, Jasmin Köffer, Ulrike Betz, Janina Koglin, Elvira Richter

**Affiliations:** 1 Labor Limbach, Heidelberg, Germany; The University of North Carolina at Chapel Hill School of Medicine, Chapel Hill, North Carolina, USA

**Keywords:** *Mycobacterium tuberculosis*, TB-PCR, MDR, drug resistance, mutations

## Abstract

The aim of this study was to evaluate the performance of the automated cobas MTB-Real-Time PCR assay for the rapid direct detection of *Mycobacterium tuberculosis* complex (MTBC) in clinical specimens and the ability of the cobas MTB-RIF/INH assay to correctly detect drug resistance to rifampin (RIF) and isoniazid (INH). The PCR assays were set up on the automated Cobas 6800 system, and the results were compared to liquid culture using BACTEC mycobacteria growth indicator tubes 960 TB system as the gold standard and line probe assays or sequencing results. A total of 500 N-acetyl-L-cysteine/sodium hydroxide (NALC-NaOH)-processed sputum samples were tested with the respective methods. The performance of MTBC detection in pulmonary specimens showed 91.8% sensitivity and 99.3% specificity in comparison to culture. The sensitivity for acid-fast bacteria (AFB) smear-positive specimens and for AFB smear-negative specimens was 100% and 85.1%, respectively. Due to the low prevalence of tuberculosis (TB) resistance in Germany, a collection of resistant TB strains with a wide variety of mutations was analyzed. The cobas MTB-RIF/INH assay detected 19 out of 21 INH-resistant and 22 out of 24 RIF-resistant TB strains. In conclusion, the cobas MTB and the cobas MTB-RIF/INH assays implemented on the automated cobas6800 instrument are reliable and versatile tools for the detection of MTB and RIF/INH resistance.

**IMPORTANCE:**

Our manuscript addresses the WHO recommendation for the use of “moderate-complexity automated NAATs for detection of TB and resistance to rifampicin and isoniazid” as a part of the WHO End TB Strategy. Rapid detection of tuberculosis (TB) patients is essential to preventing TB transmission and finally reducing TB burden. In this study, we present data on the sensitivity and specificity of the novel cobas MTB assay for TB detection in a low-incidence country, demonstrating highly promising results. Additionally, by analyzing TB strains with various mutations conferring resistance to INH and/or RMP, we assess the opportunities and limitations of the cobas MTB-RIF/INH assay in reliably detecting drug resistance in sputum specimens, thereby facilitating the early onset of appropriate treatment.

## INTRODUCTION

Tuberculosis (TB) is the world’s second leading cause of death from a single infectious agent following COVID-19. In 2022, an estimated 10.6 million people worldwide developed TB, resulting in 1.3 million deaths (1). In Germany, 4,076 cases were notified in the year 2022, corresponding to a TB incidence of 4.9 per 100,000 population (2). Until 2021, approximately 2.5% to 3% of TB cases were identified as multidrug-resistant (MDR) TB strains, increasing to 5.7% in 2022 (2). The rapid spread of multidrug-resistant (MDR) TB strains worldwide poses a major threat to health-care institutions and necessitates the urgent adoption of improved control measures. One of the WHO’s priorities in ending the tuberculosis epidemic is the implementation of rapid diagnostic tests for the accurate detection of the respective *Mycobacterium tuberculosis* complex (MTBC) pathogens (3). In addition, the availability of molecular diagnostic methods capable of directly detecting MTBC in clinical samples and simultaneously determining drug resistance facilitates the identification of patients with active MDR-TB and the implementation of appropriate drug therapies (4).

The aim of this study was to evaluate the performance of the cobas MTB-Real-Time PCR assay for the rapid and direct detection of MTBC in clinical specimens in a low-incidence country. Additionally, the study assessed the ability of the cobas MTB-RIF/INH assay to accurately detect drug resistance to rifampin (RIF) and isoniazid (INH) using a panel of *M. tuberculosis* strains with selected resistance-conferring mutation. The results of the PCR assays were compared to liquid culture using the BACTEC MGIT 960 TB system as the gold standard and line probe assay (LPA) or sequencing results, as well as phenotypic drug susceptibility testing (DST).

## MATERIALS AND METHODS

### Processing of mycobacterial samples

The study was performed at the Laboratory Limbach Heidelberg in Germany. For the study, 500 NALC-NaOH-processed sputum samples were used. All samples included in this study were de-identified leftover residual samples from standard of care testing, collected within 6 months before the start of the study. All samples were anonymized by a person not involved in the study. For all specimens, direct fluorescent smear microscopy (Auramine staining) was performed. All samples were decontaminated with NALC/ NaOH solution. Finally, 500 µL of the phosphate-buffered suspension was used for inoculation of a liquid culture (BACTEC MGIT 960; BD, Sparks, Maryland, US) and 100 µL each for inoculation on two solid media (Löwenstein-Jensen, Stonebrink, Artelt-Enclitt, Borna, Germany).

Positive cultures were analyzed by smear microscopy (Kinyoun staining method) and inoculated on a blood agar plate to detect/exclude contamination. Species identification in culture-positive specimens with acid-fast bacilli was performed using line probe assays (GenoType MTBC and GenoType Mycobacteria CM/AS) (Bruker/Hain, Nehren, Germany). In case of detection of *M. tuberculosis* complex isolates, drug susceptibility testing for RIF and INH was performed with BACTEC MGIT 960 using 0.5 µg/mL RIF and 0.1 µg/mL INH as standard critical concentrations. The underlying molecular mechanism associated with phenotypic resistance to INH and RIF was confirmed by molecular analysis of the relevant genes (GenoType MTBDR, Bruker/Hain; Sanger sequencing). All GenoType assays were performed according to the manufacturer’s instructions.

### Detection of drug resistance

In Germany, the prevalence of TB resistance is low. Therefore, the performance of the cobas MTB-RIF/INH test in detecting resistance-associated mutations was evaluated using a panel of *M. tuberculosis* strains with selected resistance-conferring mutations in *rpo*B, *kat*G, and *inh*A gene regions. The strains were obtained from the standard of care diagnostic laboratory and derived from patients notified in Germany. The strains were selected to challenge the system with a broad spectrum of mutations. The MTBC strains were characterized by LPA, Sanger sequencing, and phenotypic drug susceptibility in advance. Strains grown on LJ medium were diluted in NaCl solution at a concentration of 10e3 CFU/mL using the Cobas MTB assays.

### Cobas MTB procedure

For the preparation and setup of the cobas MTB and the cobas MTB-RIF/INH assays, 0.4 mL of the NALC-NaOH pre-treated sample was transferred to a 5 mL polypropylene tube containing 2 mL of cobas Microbial Inactivation Solution. After vortexing for 30–60 s, the NALC-NaOH-treated samples and the culture suspensions were incubated for at least 60 min at room temperature (22℃–25°C) and then vortexed for 30 s. Then, the samples were sonicated using a tube sonicator, centrifuged for 1 min at 3,000 × *g* and directly transferred into the fully automated cobas 6800 instrument (Roche). The cobas MTB-RIF/INH assay on the Cobas 6800 system was performed according to the manufacturer’s instructions.

### Statistical analysis

The data generated with the cobas MTB RIF/INH test were classified into four categories: true positive, false positive, true negative, and false negative using MTB culture results as the gold standard. Data analysis included calculation of sensitivity, specificity, and the two-sided 95% CI using 2 × 2 contingency tables. Statistic calculation was performed with the MedCalc software (www.medcalc.org).

## RESULTS

In this study, the performance of the cobas MTB for the rapid direct detection of MTBC in clinical respiratory specimens and the ability of the cobas MTB-RIF/INH to correctly predict drug resistance to RIF and INH was evaluated and compared to mycobacterial culture and phenotypic drug susceptibility testing.

A total of 500 pulmonary specimens from patients with a request for standard of care mycobacterial analysis were examined using the cobas MTB assay. Forty-nine specimens were culture positive for MTB, of which 45 showed congruent positive results with the cobas MTB assay. Among these, 22 were AFB smear positive and 23 AFB smear negative. Four specimens were MTB culture positive but negative in the PCR assay. AFB smear showed a negative result for these four cases. Of the 451 MTB culture-negative specimens, 3 yielded positive PCR results with Ct values >32. Five samples initially showed invalid results (internal control negative) but were valid upon retesting.

The performance of MTBC detection in pulmonary specimens showed a sensitivity of 91.8% and a specificity of 99.3%. Sensitivity for AFB smear-positive specimens and for AFB smear-negative specimens was 100% and 85.1%, respectively (Table 1). The average Ct values of the PCR-positive specimens were 29.5 (± 2.9; 22.5–34.4) and 28.8 (± 3.2; 22.5–34.4) for the smear-positive and 30.8 (± 1.8; 27.9–33.4) for the smear-negative specimens, respectively.

**TABLE 1:** Performance of the cobas MTB test on the Cobas 6800 system in comparison to reference MTB culture (*n* = 500, *n* = 49 culture positive)

Specimens	cobas MTB					
	TP^*a*^	FP^*a*^	TN^*a*^	FN^*a*^	% Sensitivity (95% CI)	% Specificity (95% CI)
All (*n* = 500)	45	3	448	4	91.8 (80.4–97.7)	99.3 (98.1–99.9)
Smear positive (*n* = 22)	22	0	0	0	100 (84.6–100.0)	n.a.^*b*^
Smear negative (*n* = 478)	23	3	448	4	85.2 (66.3–95.8)	99.3 (98.1–99.9)

^*a*^ TP, true positive; FP, false positive; TN, true negative; FN, false negative.

^*b*^ n.a., not applicable.

The cobas MTB-RIF/INH test for the detection of RIF and INH resistance was performed only on specimens positive for MTB with the cobas MTB assay. The results were compared to phenotypic DST. For 49 specimens positive with the cobas MTB assay, cobas MTB-RIF/INH and phenotypic DST were performed. Three specimens yielded invalid results in the cobas MTB-RIF/INH assay. These invalid specimens had a positive result with a ct value >33 for MTB detection in the preceding cobas MTB run. Presumably, the DNA concentration was too low for the analysis of the resistance genes with the cobas MTB-RIF/INH assay. The remaining 46 specimens used for evaluation showed a concordant susceptible result between the phenotypic and the molecular methods, for both INH and RIF.

To evaluate the ability of the cobas MTB-RIF/INH assay to detect different mutations responsible for resistance to INH and RIF, a collection of 34 TB strains with different well-described mutations in the *rpo*B, *inh*A, or *kat*G genes was analyzed (Table 2).

**TABLE 2:** Performance of the cobas MTB RIF/INH assay for the detection of isoniazid- and rifampicin-resistance-associated mutations in strains with known resistance patterns

Specimen no.	Result^*a*^				
	Genotype INH	cobas MTB	Genotype RIF	cobas MTB	cobas MTB channel
MP 1959	*kat*G (S315T)	INH positive	S531L	RIF positive	RIF 2
MP 2104	*inh*A (c-15t)	INH positive	WT	RIF negative	
MP 2119	WT	INH negative	S531L	RIF positive	RIF 2
MP 2252	*inh*A (c-15t)	INH positive	WT	RIF negative	
MP 2482	*inh*A (c-15t)	INH positive	WT	RIF negative	
MP 2560	*inh*A (c-15t)	INH positive	S531L	RIF positive	RIF 2
MP 2989	WT	INH negative	L511P	RIF positive	RIF 1
MP 3012	*inh*A (c-15t) and WT	INH negative	WT	RIF negative	
MP 3058	*inh*A (c-15t)	INH positive	S531L	RIF positive	RIF 2
MP 3067	WT	INH negative	H526Y	RIF positive	RIF 2
MP 3360	*kat*G (S315T)	INH positive	WT	RIF negative	
MP 3471	*kat*G (S315T)	INH positive	WT	RIF negative	
MP 3497	*inh*A (c-15t)	INH positive	WT	RIF negative	
MP 5636	*kat*G (S315T)	INH positive	S531P	RIF positive	RIF 2
MP 6314	WT	INH negative	L511P	RIF positive	RIF 1
MP 8195	WT	INH negative	H526D	RIF positive	RIF 3
TB 1237	WT	INH negative	H526D	RIF positive	RIF 3
TB 169	*kat*G (S315T)	INH positive	D516A/L533P	RIF positive	RIF 1 + 2
TB 2427	*kat*G (S315T)	INH positive	S531L	RIF positive	RIF 2
TB 2650	WT	INH negative	S531F	RIF positive	RIF 2
TB 2920	WT	INH negative	D516Y	RIF positive	RIF 2
TB 3208	*kat*G (S315T)	INH positive	H526S	RIF positive	RIF 2
TB 3660	*inh*A (t-8c)	INH positive	S531L	RIF positive	RIF 2
TB 3974	*inh*A (t-8a)	INH positive	WT	RIF negative	
TB 4049	WT	INH negative	L511P	RIF positive	RIF 1
TB 4562	*kat*G (S315T)	INH positive	D516V	RIF positive	RIF 1
TB 6466	WT	INH negative	L533P	RIF positive	RIF 2
TB 7276	WT	INH negative	L533V	RIF negative	RIF negative
TB 7773	*kat*G (S315T)	INH positive	H526G/L511P	RIF positive	RIF 1 + 3
TB 8585	*inh*A (c-15t)	INH positive	WT	RIF negative	
TB 8689	WT	INH negative	H526Y	RIF positive	RIF 2
TB 9003	*kat*G (S315T)	INH positive	S531Y	RIF positive	RIF 1
TB 9212	WT	INH negative	T508A	RIF negative	RIF negative
TB 9696	*inh*A (−15 region)	INH negative	WT	RIF negative	

^*a*^ INH, isoniazid; RIF, rifampicin; WT, wild-type sequence.

Of the 21 strains carrying a clinically relevant INH resistance mutation, 19 were correctly identified as positive (resistant) with the assay (Table 2), while the remaining 13 INH-susceptible isolates were correctly identified as INH negative. One of the two isolates not detected with the assay contained a mixture of wild-type (susceptible) and mutated (resistant) strains based on the LPA results. The second isolate, which was not recognized as INH resistant by the COBAS assay, had a not further characterized mutation in the *inh*A −15 region (but not c-15 as excluded by LPA). Both strains were phenotypically INH resistant.

Of the 24 strains carrying 1 or 2 mutations in the *rpo*B gene, 22 were correctly identified as RIF positive (resistant) by the assay (Table 2). Ten strains without *rpo*B mutations were correctly identified as RIF negative (susceptible). One isolate which was not detected as RIF resistant had a *rpo*B T508A (=T427A) mutation, and the second isolate not detected as resistant carried a *rpo*B L533V (=L452V) mutation.

## DISCUSSION

TB remains a significant public health concern worldwide. Early and reliable detection is crucial to reducing morbidity, mortality, and transmission. The important role of molecular diagnostic tests in this context has been well established (3).

In this study, we evaluated the diagnostic performance of the cobas MTB assay on the automated Cobas 6800 systems for the detection of MTB in processed sputum specimens in comparison to mycobacterial culture. The molecular assay showed an overall sensitivity of 91.8% (95% CI, 80.4% to 97.7%) and a specificity of 99.3% (95% CI, 98.1% to 99.9%). These data are comparable to, and even better than, the performance of other systems using processed sputum specimens, including Xpert MTB/RIF (Cepheid, Sunnyvale, CA, USA), Anyplex MTB/NTMe (Seegene, Seoul, Korea), the RealTime MTB (Abbott, Des Plaines, IL, USA), Fluorotype MTB, FluoroType MTBDR (both Hain Lifescience/Bruker), and the BD MAX MDR-TB (Becton, Dickinson and Company, Sparks, MD) (5–16). To date, two studies have been published evaluating the performance of the cobas MTB assay on the Cobas 6800 (16, 17). The authors reported an overall sensitivity of 89.2% (95% CI, 81.7% to 93.9%) and 94.7% (95% CI, 88.1% to 98.3%) for decontaminated specimens, which is similar to the results reported in this study. The sensitivity for smear-negative, culture-positive specimens was reported as 81.8% (95% CI, 59.7% to 94.8%) and 63.0% (95% CI, 44.2% to 78.5%) in these studies, respectively. In our study, the cobas MTB test demonstrated an increased sensitivity for the detection of MTB in smear-negative, culture-positive specimens (85.2% [95% CI, 66.3% to 95.8%]). This performance is comparable or at least superior to those reported in other studies. Therein, the authors published sensitivities ranging from 22% to 91.8% for the platforms mentioned above (References above). High accuracy in smear-negative culture-positive specimens is relevant considering the delayed results of MTB-positive culture, thus leading to unidentified TB transmission. In our study, four cases were PCR negative and culture positive. For these false-negative cases, culture showed extended time of detection (17–35 days to positivity) of these specimens, reflecting the paucibacillary nature of these specimens. In a study evaluating the analytical sensitivity of several MTB PCR assays, the PCR cobas MTB showed an LOD_95_ similar to WHO-recommended molecular MTB assays, including the widely used Xpert MTB/RIF test (18).

The specificity of cobas MTB in our study was 99.3%, with 3 false-positive PCR results in 452 culture-negative specimens. These specimens showed high Ct values (33–37), potentially indicating the detection of DNA from non-viable bacilli in these specimens. Unfortunately, the final medical history was unavailable for these patients.

In this study, the cobas MTB RIF/INH resistance assay was also evaluated in MTB-positive specimens. The cobas MTB RIF/INH is considered a reflex test or can be set up in parallel to the cobas MTB in high-incidence settings. Both key antitubercular drugs, RIF and INH, are tested. Additional INH testing is highly warranted due to a considerable rate of mono-INH-resistant strains even in low-incidence countries like Germany (2). In our low-prevalence study setting, only drug-susceptible clinical samples were available, which all were correctly identified by the molecular assay compared to DST. Nadarajan et al. (17) tested a higher number of samples using specimens from Sierra Leone and Germany. They demonstrated a sensitivity of 88.4% (95% CI, 75.5% to 94.9%; 38/43 samples) for detection of RIF resistance and a sensitivity of 76.6% (95% CI, 62.8% to 86.4%; 36/47 samples) for detection of INH resistance. Analysis using whole-genome sequencing showed that the discordant results for RIF and INH resistance were mainly due to uncommon mutations in samples from Sierra Leone. The authors corroborated the importance of geographical variations for detection of resistance-conferring mutations.

In this study, we additionally analyzed a collection of resistant TB strains with a wide variety of *rpo*B, *inh*A, and *kat*G mutations to ensure the inclusion of even rare mutations. The COBAS assay detected 19 out of 21 INH-resistant and 22 out of 24 RIF-resistant TB strains. One INH-resistant isolate exhibited heteroresistance, with both wild-type and mutant strains present, as revealed by LPA results. Detection of a resistant subpopulation can be challenging for molecular assays. The second undetected strain harbored a highly uncommon mutation, according to the WHO catalogue of mutations (19), which is presumably not covered by the assay probes. Both *rpo*B mutations (T508A (=T427A), L533V (=L452V)) not detected with the assay are also very rare. Moreover, according to the WHO catalogue (19), the *rpo*B T508A (=T427A) mutation may not confer resistance (19). Phenotypically, both strains were susceptible to RIF, with minimum inhibitory concentration (MIC) values ≤0.125 µg/mL. Presumably, for these rare mutations, no probes are present in the cobas RIF/INH assay, possibly due to their unclear clinical significance. No strain carrying the D516F (D435F) mutation, which was reported as negative in the evaluation by de Vos et al. (18), was included in our study.

Positive (resistant) results were obtained for strains carrying so-called borderline mutations (L511*P* = L430P; D516Y = D435Y; H526S = H445S; L533*P* = L452P); this aligns with the WHO catalogue (19). However, these strains were phenotypically susceptible to RIF, with MIC values ranging from 0.125 to 0.5 µg/mL. The differentiation of these discrepancies in a laboratory can only be enabled by sequence analysis of the *rpo*B gene.

The remaining 15 strains with concordant RIF-resistant results all carried mutations associated with phenotypic resistance, including a strain with the rpoB S531F (S450F) mutation, which was tested negative in a previous evaluation of the cobas assay (17). Overall, the assay performed very well in detecting both INH and RIF resistance.

In the cobas assay, RIF-positive (resistant) results are displayed in three different channels. Even different amino acid exchanges at the same amino acid position are displayed in different channels in some cases. Consequently, it is not possible to infer a specific amino acid position from the cobas result, which would be very helpful in cases with RIF borderline mutation to be aware of a different phenotypic result. Similarly, for INH, no conclusion can be drawn on the affected gene since all results, independent of *kat*G or *inh*A mutation, are displayed in the same channel.

The cobas MTB RIF/INH assay evaluated in this study was set up on the fully automated Cobas 6800 system. Basically, the instrument is a high-throughput system designed to process large batches of up to 384 samples within an 8 hr shift, with the capability to perform multiple tests in parallel (4). Especially during the SARS-CoV-2 pandemic, the system was an important tool in processing a high volume of specimens in a short time (20). The workflow of the PCR assay and the handling of the instrument are straightforward: after an inactivation step and sonication, the sample tube is directly loaded onto the system on which DNA extraction, amplification, detection, and result generation are performed automatically. The total time to result, including DNA extraction, of 2 h and 30 min is comparable to other platforms like FluoroType MTBDR (Bruker/Hain), BD Max MDRTB (BD) with estimated 3 h and 41 min for both assays (18). While the Cobas 6800 and the Cobas 8800 instruments are suitable for the use in medium to big central core laboratories, the recently introduced smaller Cobas 5800 system can be utilized in smaller laboratories. In conclusion, the cobas MTB assay represents a promising tool in the detection of MTB and the RIF/INH resistance across various laboratory settings.
